# Advanced glycation endproducts and their receptor in different body compartments in COPD

**DOI:** 10.1186/s12931-016-0363-2

**Published:** 2016-04-26

**Authors:** Susan J. M. Hoonhorst, Adèle T. Lo Tam Loi, Simon D. Pouwels, Alen Faiz, Eef D. Telenga, Maarten van den Berge, Leo Koenderman, Jan-Willem J. Lammers, H. Marike Boezen, Antoon J. M. van Oosterhout, Monique E. Lodewijk, Wim Timens, Dirkje S. Postma, Nick H. T. ten Hacken

**Affiliations:** Department of Pulmonary Diseases, University of Groningen, University Medical Center Groningen, Hanzeplein 1, 9713 GZ Groningen, The Netherlands; University of Groningen, University Medical Center Groningen, GRIAC research institute, Groningen, The Netherlands; Department of Respiratory Medicine, University Medical Center Utrecht, Utrecht, The Netherlands; Department of Epidemiology, University of Groningen, University Medical Center Groningen, Groningen, The Netherlands; Department of Pathology and Medical Biology, University of Groningen, University Medical Center Groningen, Groningen, The Netherlands

**Keywords:** COPD, RAGE, Advanged glycation end-products, sRAGE

## Abstract

**Background:**

Chronic obstructive pulmonary disease (COPD) is a chronic lung disease characterized by chronic airway inflammation and emphysema, and is caused by exposure to noxious particles or gases, e.g. cigarette smoke. Smoking and oxidative stress lead to accelerated formation and accumulation of advanced glycation end products (AGEs), causing local tissue damage either directly or by binding the receptor for AGEs (RAGE). This study assessed the association of AGEs or RAGE in plasma, sputum, bronchial biopsies and skin with COPD and lung function, and their variance between these body compartments.

**Methods:**

Healthy smoking and never-smoking controls (*n* = 191) and COPD patients (*n* = 97, GOLD stage I-IV) were included. Autofluorescence (SAF) was measured in the skin, AGEs (pentosidine, CML and CEL) and sRAGE in blood and sputum by ELISA, and in bronchial biopsies by immunohistochemistry. eQTL analysis was performed in bronchial biopsies.

**Results:**

COPD patients showed higher SAF values and lower plasma sRAGE levels compared to controls and these values associated with decreased lung function (*p* <0.001; adjusting for relevant covariates). Lower plasma sRAGE levels significantly and independently predicted higher SAF values (*p* < 0.001). One SNP (rs2071278) was identified within a region of 50 kB flanking the AGER gene, which was associated with the gene and protein expression levels of AGER and another SNP (rs2071278) which was associated with the accumulation of AGEs in the skin.

**Conclusion:**

In COPD, AGEs accumulate differentially in body compartments, i.e. they accumulate in the skin, but not in plasma, sputum and bronchial biopsies. The association between lower sRAGE and higher SAF levels supports the hypothesis that the protective mechanism of sRAGE as a decoy-receptor is impaired in COPD.

**Electronic supplementary material:**

The online version of this article (doi:10.1186/s12931-016-0363-2) contains supplementary material, which is available to authorized users.

## Background

Chronic obstructive pulmonary disease (COPD) is characterized by chronic airflow limitation, accompanied by persistent inflammation of the airways, mainly caused by cigarette smoking. Both smoking and inflammation are associated with oxidative stress leading to accelerated formation and accumulation of advanced glycation end products (AGEs) [1, 2].

AGEs are a heterogeneous and complex group of compounds that are irreversibly formed by non-enzymatic glycation and oxidation of proteins and lipids [3]. The formation of AGEs accumulates in tissues with ageing. Furthermore, under oxidative stress and inflammatory conditions their formation and accumulation increase. Therefore, accumulation of AGEs can be used as a read-out system for exposure to oxidative stress during life. This is particularly true in tissues with slow turnover, more than in tissues or products from tissues with rapid turnover. The best known AGEs are N^ε^-(carboxymethyl)lysine (CML), N^ε^-(carboxyethyl)lysine (CEL) and pentosidine. AGEs cause local tissue damage by affecting protein structure, by formation of crosslinks between molecules, or by binding the receptor for AGEs (RAGE) [4, 5]. RAGE is a member of the immunoglobulin superfamily and is a pattern-recognition receptor on cell surfaces. Ligation of RAGE triggers inflammatory responses, induces oxidative stress, and in turn causes RAGE over-expression. This may finally leads to increased tissue remodeling [5]. Interestingly, expression of RAGE in the lung has shown to be relatively high when compared with other tissues [6]. The gene encoding RAGE, AGER, has been shown to be a susceptibility gene for lung function decline and the onset of COPD [7, 8]. Previously, a number of Single Nucleotide Polymorphism (SNP)s within and in close proximity to the *AGER* gene have been identified to play an important role in its transcriptional and translation regulation (PMID: 23886569). Furthermore, it has been shown that immunostaining of the receptor is increased in bronchial biopsies and lung parenchyma of COPD patients [9, 10]. Next to AGEs, RAGE can also be activated by endogenous danger signals, or damage associated molecular patterns (DAMPs). Several of these RAGE activating DAMPs, e.g. HMGB1, S100A9 and LL-37 have been shown to be increased in lung fluid or serum from COPD patients compared to smoking and non-smoking controls [11, 12]. Importantly, RAGE also exists as soluble form (sRAGE). It has been postulated that sRAGE can act as a decoy receptor by clearance of circulating AGEs, preventing ligation of membrane bound RAGE. This possible ‘protective’ mechanism may be reduced in COPD, as levels of sRAGE have found to be lower in COPD patients than in non-COPD controls [13–17].

A few studies have indicated that AGEs are involved in the pathology of COPD. One study showed increased accumulation of AGEs in lung parenchyma and small airways of COPD patients [9]. We and others found increased AGE accumulation in the skin of COPD patients compared to healthy smoking and never-smoking controls [18, 19]. Furthermore, plasma CML levels in COPD are elevated compared to non-COPD controls [19], suggesting a systemic component that may contribute to AGE accumulation outside the lung, and to extra-pulmonary manifestations of COPD.

Taken together, studies so far suggested that the AGE-RAGE axis is involved in the pathophysiology of COPD. In the current study we evaluated both AGEs and sRAGE levels in plasma, sputum, bronchial biopsies and the skin in the same study subjects. Young (18–40) and old (40–75) smokers and never-smokers, and mild-to-very severe COPD patients were included. We studied whether the expression of AGEs or RAGE in the different tissues was associated with COPD and lung function values, and whether the expression of AGEs was associated with the levels of RAGE in different tissues. Lastly, with a cis-eQTL analysis we investigated the association of SNPs flanking the *AGER* gene with skin autofluorescence and serum sRAGE levels.

## Methods

### Subjects, ethics, consent and permissions

Data were collected from two studies performed in Groningen and Utrecht, the Netherlands (Clinicaltrials.gov: NCT00807469 and NCT00848406 (A multi-center study [20]) and NCT00848406). All participating subjects gave peripheral blood, bronchial brushings and performed an AGE-reader measurement, while a subgroup of subjects underwent sputum induction and bronchoscopy with collection of bronchial biopsies. All measurements were obtained by using standardized protocols. All studies, sample collection and genetic studies were approved by the medical ethics committees of University Medical Centers Groningen (UMCG) and Utrecht (UMCU), the Netherlands. All participants gave their written informed consent.

Mild to very severe COPD patients (40–75 years, >10 packyears), as classified by the Global Initiative for Chronic Obstructive Lung Disease (GOLD) were recruited from outpatient clinics of UMCG and UMCU. Old (40–75 years) and young (18–40 years) healthy smokers and healthy never-smokers were recruited by advertisements. Old smokers had a smoking history >10 packyears and young smokers >0.5 packyears. All never-smoking subjects had smoked <0.5 packyears. Healthy participants had no history of pulmonary diseases and showed normal spirometry. Exclusion criteria for all groups were alpha-1 antitrypsin deficiency and a doctors’ diagnosis of asthma.

### Determination of AGEs and RAGE in peripheral blood samples and sputum

Blood was collected in tubes containing EDTA and was immediately placed on ice. After centrifugation (twice at 2000 rcf_max_, 10 min, 4 °C) samples were stored at -80 °C until analysis. Sputum induction was performed according standard protocols (detailed methods in Additional file 1). Sputum samples were centrifuged (10 min, 450 g, 4 °C) and the supernatant was stored at -80 °C until analysis.

In plasma and sputum samples, ELISA was performed to determine levels of total sRAGE (cleaved and secreted forms) (RAGE DuoSet; R&D Systems, Minneapolis, MN, USA), CEL (Cell Biolabs Inc. San Diego, CA, USA), CML (Cell Biolabs Inc. San Diego, CA, USA) and Pentosidine (Uscn Life Science Inc., Wuhan, China), all according to the manufacturer’s instructions.

### Determination of AGEs and RAGE in bronchial biopsies

Bronchial biopsies were taken from subsegmental carinae of the right lower lobe. Biopsies were fixed in 4 % neutral buffered formalin, processed and embedded in paraffin and cut in 3 μm sections. After antigen retrieval, sections were incubated with the primary monoclonal antibody against AGEs (anti-AGEs (clone 6D12), 1:750, Cosmo Bio Co, Ltd, Tokyo, Japan) or RAGE (anti-RAGE (ab7764), 1:1500, Abcam, Cambridge, UK). Immunohistochemical stainings were performed using the DAKO autostainer (DAKO, Glostrup, Denmark). Quantification of both stainings was performed by calculating the percentage positive and strong positive pixels of the total amount of pixels in whole biopsies, using ImageScope (Aperio Technologies, version 11.2.0.780). Detailed immunohistochemistry and quantification procedures are presented in the Additional file 1.

### Measurement of AGEs using Skin autofluorescence in the skin

Skin autofluorescence (SAF) was assessed non-invasively by the AGE-Reader™ (DiagnOptics B.V., Groningen, The Netherlands) [21]. Technical details of this device have been extensively described elsewhere and briefly in the Additional file 1 [22]. In short, the volar surface of subject’s forearm was positioned on top of the device and three consecutive measurements were performed for each subject. In all analyses, SAF was expressed as the mean of these three measurements in arbitrary units (AU).

### Gene expression analysis

We assessed whether cis-acting SNPs influence the gene and protein expression of AGER and RAGE, respectively. DNA samples were genotyped with Illumina Human CytoSNP 12 and Illumina OmniExpress Exome. SNPs genotyped in both arrays were select for the eQTL analysis. Gene expression profiles were obtained from bronchial brushing using a Human genome ST v1.0 arrays. Linear regression analysis was used to test for association between the SNPs and 2-log transformed gene expression levels and soluble RAGE measured in sputum and plasma. SNPs were tested in an additive genetic model and the models were adjusted for gender, smoking status and age. A cis-eQTL was defined as a SNP that was significantly associated with gene or protein expression within a 50 kB distance of the AGER gene.

### Statistical analysis

Differences in expression of AGEs and RAGE between groups were analyzed by Kruskal-Wallis test, followed by Mann-Whitney U tests if significant. Associations with COPD were examined by multiple regression analyses with AGEs or RAGE expression as dependent variables, and COPD or lung function values as predictor variables. Associations of AGEs and RAGE between different compartments were additionally analyzed by multiple regression models. All models were adjusted for co-variates that associate with AGE formation, including age, gender, packyears, BMI, LDL cholesterol, and triglycerides. Benjamini Hochberg corrections were applied to correct for multiple testing. Regression models were considered valid if the residuals were normally distributed. Statistical analyses were performed using the statistical program IBM SPSS Statistics version 20.

## Results

### Subject characteristics

In total, 108 young controls (including 36 never-smokers and 72 smokers), 83 old controls (including 28 never-smokers and 55 smokers) and 97 COPD patients (32 GOLD I, 25 GOLD II, 24 GOLD III, 16 GOLD IV) were included. Group characteristics are presented in Table 1.

**Table 1:** Group characteristics

	Young healthy	Old healthy	COPD
	*n* = 108	*n* = 83	*n* = 97
Age, years	25 (66)	54 (8.9)	62 (7.6)
Males, n (%)	54 (50)	60 (72)	68 (70)
Current smokers, n (%)	72 (67)	54 (65)	51 (53)
Cigarettes per day	9 (6.8)	16 (7.1)	11 (7.9)
Packyears	3.1 (4.8)	20 (18.0)	38 (16.8)
BMI, kg/m^2^	23.0 (2.8)	25.3 (3.6)	25.5 (4.7)
FEV_1_, %pred	108 (9.5)	110 (13.5)	62 (27)
FEV_1_/FVC, %	85 (5.7)	79 (5.0)	48 (14)
RV/TLC, %	23.3 (5.1)	31.1 (4.4)	45.8 (11.3)
FEF_25-75_, %pred	101 (18.7)	100 (30.4)	24 (17)
DLCOc/VA, %pred	97 (13.3)	98 (12.6)	67 (24)
DLCO %pred	91.6 (12.0)	90.0 (12.3)	61.6 (23.6)
LDL cholesterol, mmol/L	2.6 (0.8)	3.6 (1.0	3.4 (1.0)
Triglycerides, mmol/L	1.0 (0.8)	1.4 (1.0)	1.2 (0.7)
Fasting glucose, mmol/L	5.2 (1.3)	5.6 (0.7)	5.8 (0.7)
Creatinine, μmol/L	73.7 (10.9)	80.2 (14.5)	82.7 (14.8)

Data is presented as mean (sd) unless otherwise stated

*n* number, *BMI* body mass index, *FEV*_*1*_ forced expiratory volume in one second, *FVC* forced expiratory volume, *RV* residual volume, *TLC* total lung capacity, *FEF* forced expiratory flow, *DLCO(*_*c*_ */VA)* diffusion coefficient for carbon monoxide, *LDL* low density lipoprotein

### AGEs

**Fig. 1:**
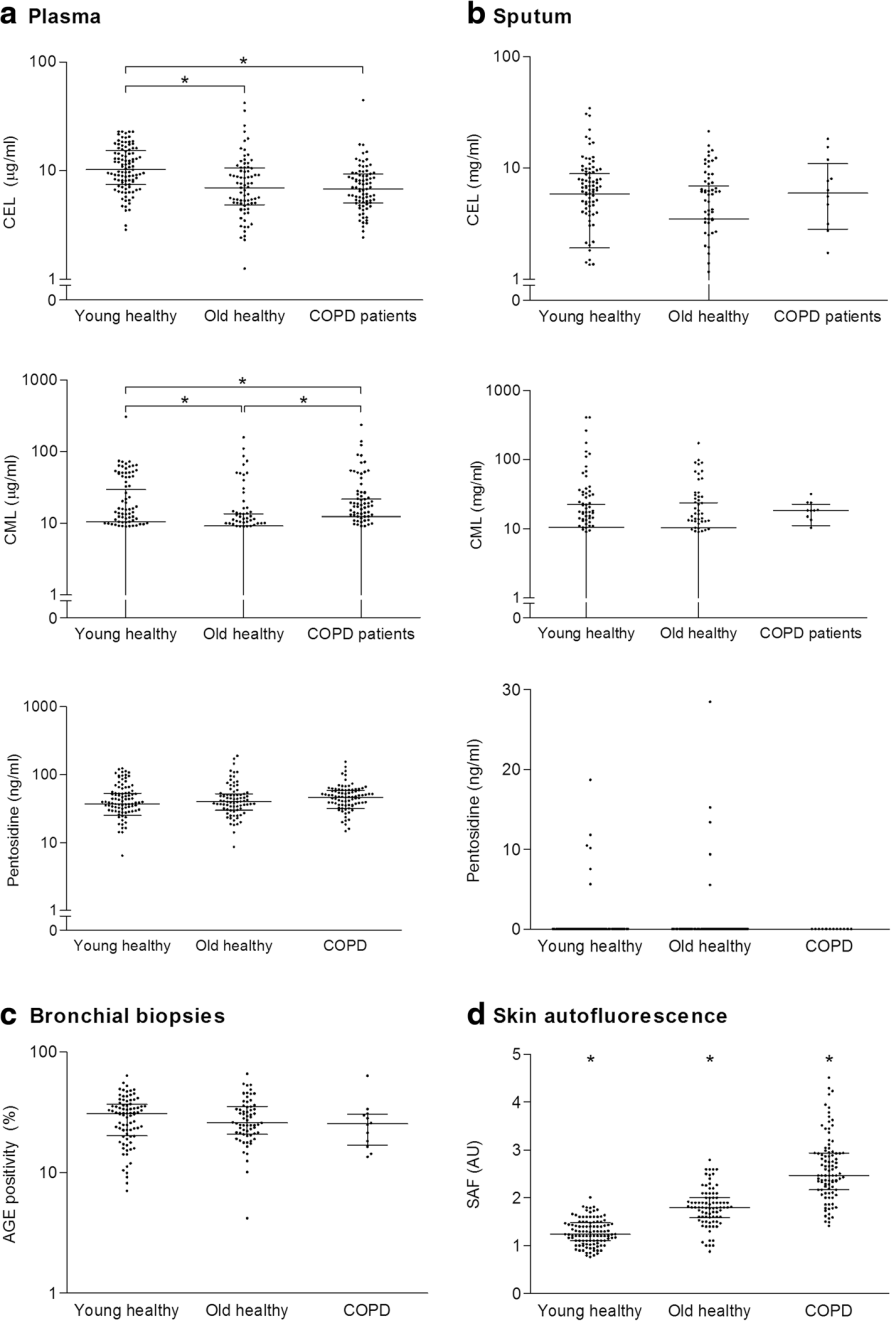
The expression of AGEs in plasma, sputum, bronchial biopsies and the skin. The levels of AGEs in **a** plasma, **b** sputum, **c** bronchial biopsies and **d** skin (SAF). Horizontal lines represent median values with interquartile ranges, * *p* < 0.05 between groups

Expression of AGEs in plasma, sputum, bronchial biopsies and the skin is presented in Fig. 1 and Table 2.

**Table 2:** AGE and RAGE expression in young and old subjects, and COPD patients

	Young healthy	Old healthy	COPD GOLD I-IV	Kruskal-Wallis
	*<40 years*	*>40 years*	*>40 years*	*p-value*
Plasma	*n = 105*	*n = 82*	*n = 95*	
CEL, μg/ml	10.2 (7.4-15.4)	7.0 (4.8-10.6) **	6.8 (5.1-9.4) **	0.000*
CML, μg/ml	10.5 (0.0-29.5)	9.2 (0.0-13.5) **	12.5 (0.0-21.6) **^a^	0.042*
Pentosidine, ng/ml	36.8 (25.3-53.1)	39.9 (30.1-52.0)	46.1 (31.8-58.5)	0.181
sRAGE, pg/ml	795 (614-1089)	805 (617-1032)	414 (292-592) **^a^	0.000*
Induced sputum	*n = 97*	*n = 73*	*n = 12*	
CEL, μg/ml	5.9 (1.9-8.9)	3.50 (0.0-6.9)	6.0 (2.9-11.0)	0.064
CML, μg/ml	10.6 (0.0-22.7)	10.4 (0.0-23.7)	18.3 (11.2-22.5)	0.409
Pentosidine, ng/ml	0.0 (0.0-0.0)	0.0 (0.0-0.0)	0.0 (0.0-0.0)	0.650
sRAGE, pg/ml	78.1 (0.0-160.2)	114.0 (0.0-240.5)	110.9 (49.7-161.8)	0.117
Bronchial Biopsies	*n = 85*	*n = 68*	*n = 12*	
AGEs, positivity (%)	31.1 (20.3-36.9)	25.9 (20.9-35.4)	25.5 (16.9-30.7)	0.418
RAGE, positivity (%)	9.8 (6.5-15.1)	8.2 (5.7-11.8)	8.0 (4.2-11.4)	0.204
Skin	*n = 107*	*n = 83*	*n = 96*	
AGE-reader, SAF	1.2 (1.1-1.5) *	1.8 (1.6-2.0) *	2.5 (2.2-2.9) *	0.000*

Data is expressed as median (IQR). * *p* < 0.05 between all groups, ** *p* < 0.05 compared with the young healthy group, ^a^ compared with the old healthy group

*CEL* N^ε^-(carboxyethyl)lysine, *CML* N^ε^-(carboxymethyl)lysine, *RAGE* receptor for advanced glycation endproducts, *AGEs* advanced glycation endproducts, *SAF* skin autofluorescence

In plasma (Fig. 1a), CEL levels were significantly higher in young healthy subjects compared to old healthy subjects and COPD patients. Furthermore, plasma CML levels were significantly higher in COPD patients compared to young and old subjects, and higher in the young group than in the old healthy group. Plasma pentosidine levels did not differ between groups. In sputum (Fig. 1b), CEL and CML levels did not differ between groups, whereas pentosidine levels were too low to be detected; only 11 sputum supernatant samples of the total of 182 samples were above the detection limit of 1.45 ng/ml. AGEs immunopositivity in whole bronchial biopsies was not differently expressed between groups, (Fig. 1c), neither were quantitative analyses in the intact and basal epithelium, smooth muscle and connective tissue (Additional file 1: Figure S1 and S2). However, accumulation of AGEs in the skin was significantly different between all groups, with highest SAF values in COPD patients and lowest values in the young group (Fig. 1d).

In all measurements, the levels of AGEs did not differ between the different stages of COPD (GOLD I-IV, Additional file 1: Table S1). Additionally, AGE levels are presented separately for healthy never-smokers and smokers (Additional file 1: Table S1).

### (s)RAGE

**Fig. 2:**
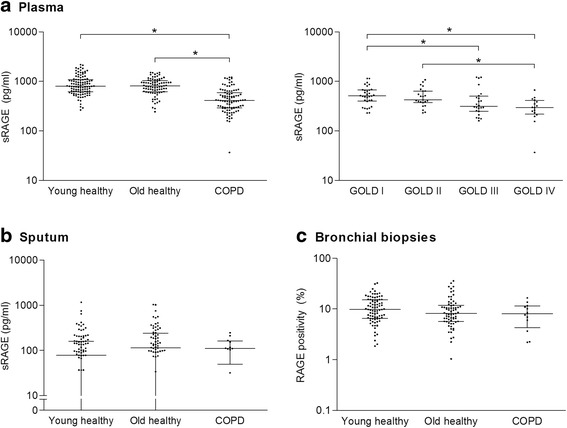
RAGE expression in plasma, sputum and bronchial biopsies. RAGE levels in **a** plasma, **b** sputum, and **c** bronchial biopsies. Horizontal lines represent median values with interquartile ranges, * *p* < 0.05 between groups

The levels of RAGE in plasma, sputum and bronchial biopsies are presented in Fig. 2 and Table 2.

**Fig. 3:**
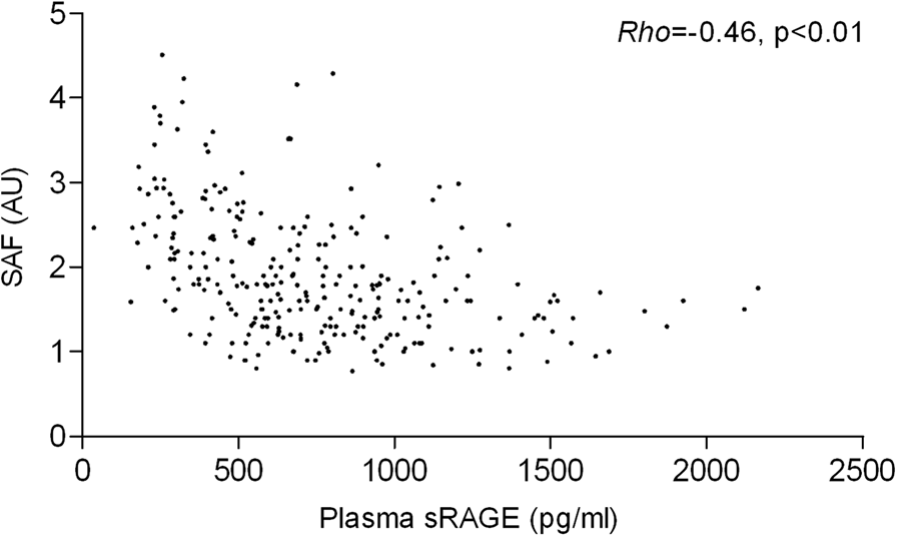
Associations between sRAGE and SAF. *Rho* = correlation coefficient, SAF = skin autofluorescence, sRAGE is soluble receptor for advanced glycation endproducts. Association after adjustment for age, gender, packyears, BMI, LDL cholesterol and triglycerides was in B = 0.00, *p* = <0.01

In plasma, sRAGE levels were significantly lower in COPD patients compared to young and old healthy subjects (Fig. 2a). In addition, COPD GOLD stage III patients showed lower sRAGE levels compared to GOLD stage I patients. Furthermore, GOLD stage IV patients showed lower sRAGE levels than GOLD stage I and II patients (Fig. 3a and Additional file 1: Table S1). No differences were found between young and old healthy subjects. RAGE levels in sputum and RAGE immunopositivity in whole sections from bronchial biopsies did not differ between groups (Fig. 2b and c). Upon studying different parts of the bronchial biopsies (intact and basal epithelium, smooth muscle, connective tissue) no group differences were found (Additional file 1: Figure S1 and S2).

RAGE levels in healthy never-smokers and smokers are presented in Additional file 1: Table S1.

### Associations between COPD, lung function, emphysema and AGEs and RAGE

Table 3 A-B shows the results of multiple regression analyses with COPD or lung function values as predictors of AGEs and RAGE expression in the different compartments.

**Table 3:** Associations of AGEs and RAGEs with COPD and lung function in the total population

A. Associations of AGEs with COPD and lung function											
Dependent variables		Predictor variables									
		COPD, n/y		FEV1, % predicted		FEV1/FVC (%)		RV/TLC (%)		FEF25-75, % predicted	
		β	*p*-value	β	*p*-value	β	*p*-value	β	*p*-value	β	*p*-value
Plasma	CEL	0.009	0.924	-0.465	0.642	0.581	0.562	0.117	0.204	0.0.41	0.635
	CML	0.093	0.327	-0.081	0.283	-0.088	0.337	0.052	0.572	-0.027	0.761
	Pentosidine	-0.035	0.711	-0.020	0.788	0.069	0.451	0.093	0.314	-0.011	0.901
Sputum	CEL	0.093	0.311	-0.139	0.101	-0.044	0.691	0.11	0.353	-0.059	0.518
	CML	0.029	0.757	-0.030	0.728	0.043	0.706	0.021	0.862	0.000	0.998
	Pentosidine	-0.025	0.792	-0.124	0.152	0.022	0.844	-0.024	0.841	-0.053	0.568
Bronchial biopsies	AGE positivity	-0.065	0.550	0.139	0.155	0.075	0.551	-0.126	0.420	0.094	0.379
Skin	SAF	**0.426**	**<0.001**	**-0.302**	**<0.001**	**-0.357**	**<0.001**	**0.265**	**<0.001**	**-0.347**	**<0.001**
B. Associations of RAGE with COPD and lung function											
Dependent variables		Predictor variables									
		COPD, n/y		FEV1, % predicted		FEV1/FVC (%)		RV/TLC (%)		FEF25-75, % predicted	
		β	*p*-value	β	*p*-value	β	*p*-value	β	*p*-value	β	*p*-value
Plasma	sRAGE	**-0.422**	**<0.001**	**0.310**	**<0.001**	**0.405**	**<0.001**	**-0.236**	**0.002**	**0.297**	**<0.001**
Sputum	sRAGE	-0.110	0.194	0.084	0.289	0.012	0.908	-0.015	0.895	-0.041	0.622
Bronchial biopsies	RAGE positivity	-0.083	0.365	-0.053	0.529	0.04	0.715	0.241	0.060	-0.038	0.678

Values in bold represent significant associations. β = standardized regression coefficient for predictor variables. Models in Table A were adjusted for age, gender, packyears, BMI, LDL cholesterol and triglycerides; models in Table B were adjusted for age, gender and packyears

*FEV*_*1*_ forced expiratory volume in one second, *FVC* forced expiratory volume, *RV* residual volume, *TLC* total lung capacity, *FEF* forced expiratory flow, *CEL* N^ε^-(carboxyethyl)lysine, *CML* N^ε^-(carboxymethyl)lysine, *AGEs* advanced glycation endproducts, *SAF* skin autofluorescence, *RAGE* receptor for advanced glycation endproducts

In established COPD, a lower FEV_1_ % predicted, FEV_1_/FVC, FEF25–75 % predicted, as well as a higher RV/TLC were associated with a higher SAF, independently of age, gender, number of packyears, BMI, LDL cholesterol and triglycerides (Table 3A). No associations were observed between COPD or lung function values on one hand and AGEs in plasma, sputum and bronchial biopsies on the other hand.

Regarding RAGE, both established COPD and impaired lung function values were associated with higher levels of sRAGE in plasma (Table 3B). In line, a weak but significant negative correlation was shown for the plasma levels of sRAGE with FEV1% predicted post bronchodilator (*r*^2^ = 0.0943, *p* = 0.0025). No associations were found of COPD or lung function values with RAGE levels in sputum and bronchial biopsies.

**Fig. 4:**
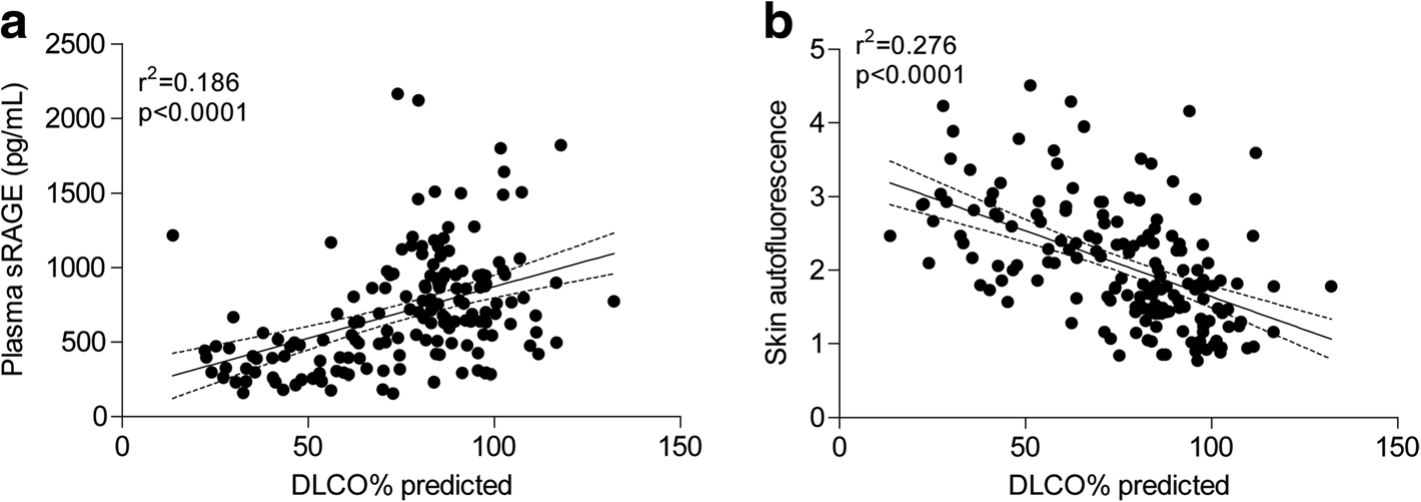
Associations of DLCO% predicted with (**a**) plasma sRAGE levels and (**b**) skin autofluorescence. All participants from the study were added, including young healthy controls, old healthy controls and COPD patients. Significance was tested using a linear regression analysis. Dotted lines indicate the 95 % confidence intervals

The percentage of the predicted diffusion capacity (DLCO% predicted) is used as a measure for the amount of emphysema (Table 1). Here, we found a positive correlation between the DLCO% predicted and the plasma levels of sRAGE (*r*^2^ = 0.186, *p* < 0.0001) and a negative correlation between the DLCO% predicted and the SAF levels (*r*^2^ = 0.276, *p* < 0.0001) (Fig. 4). These data indicate that emphysema is associated with low plasma levels of sRAGE and with high levels of accumulated AGEs in the skin.

### Associations of AGEs and RAGE expression between different compartments

Results of multiple regression analyses are presented in Table 4, reflecting associations between AGEs and RAGE expression in the different tissues after adjustment for age, gender, packyears, BMI, LDL cholesterol and triglycerides. Lower plasma sRAGE levels were significantly associated with higher SAF values.

**Table 4:** Associations between AGEs and RAGE expression in different tissues

		Plasma								Sputum								Bronchial biopsies				Skin	
		CEL		CML		Pentosidine		sRAGE		CEL		CML		Pentosidine		sRAGE		AGE positivity		RAGE positivity		SAF	
*Predictor variables:*		*B*	*p*	*B*	*p*	*B*	*p*	*B*	*p*	*B*	*p*	*B*	*p*	*B*	*p*	*B*	*p*	*B*	*p*	*B*	*p*	*B*	*p*
Plasma	CEL	---	---	0.42	0.34	-0.39	0.27	3.21	0.47	0.07	0.46	0.99	0.20	0.02	0.59	-3.70	0.21	0.43	0.08	0.28	0.04	0.00	0.91
	CML	0.01	0.34	---	---	0.11	0.07	-1.01	0.13	0.02	0.22	-0.13	0.34	0.00	0.77	0.51	0.28	0.07	0.03	0.00	0.78	0.00	0.72
	Pentosidine	-0.02	0.27	0.13	0.07	---	---	-1.84	0.01	0.04	0.01	-0.13	0.38	0.01	0.44	0.46	0.37	0.04	0.20	-0.01	0.51	0.00	0.81
	sRAGE	0.00	0.47	-0.01	0.13	-0.01	0.01	---	---	0.00	0.21	-0.01	0.57	0.00	0.46	0.04	0.30	-0.01	0.09	0.00	0.64	0.00	0.00*
Sputum	CEL	0.06	0.46	0.62	0.22	1.14	0.01	-6.92	0.21	---	---	2.93	0.00*	0.17	0.00*	-3.30	0.23	0.51	0.01	-0.07	0.53	0.00	0.91
	CML	0.01	0.20	-0.05	0.34	-0.04	0.38	-0.33	0.57	0.03	0.00*	---	---	0.00	0.50	-0.63	0.03	0.03	0.07	0.00	0.72	0.00	0.51
	Pentosidine	0.10	0.59	-0.25	0.77	0.58	0.44	6.77	0.46	0.47	0.00*	0.92	0.50	---	---	-5.94	0.21	0.12	0.70	-0.19	0.27	-0.01	0.50
	sRAGE	0.00	0.21	0.02	0.28	0.01	0.37	0.18	0.30	0.00	0.23	-0.06	0.03	0.00	0.21	---	---	0.00	0.96	0.00	0.95	0.00	0.63
Bronchial	AGE positivity	0.06	0.08	0.59	0.03	0.33	0.20	-4.99	0.09	0.14	0.01	0.92	0.07	0.01	0.70	0.09	0.96	---	---	0.10	0.07	0.00	0.39
biopsies	RAGE positivity	0.13	0.04	-0.13	0.78	-0.29	0.51	-2.52	0.64	-0.06	0.53	0.32	0.72	-0.06	0.27	-0.19	0.95	0.30	0.07	---	---	0.00	0.85
Skin	SAF	-0.10	0.91	-1.50	0.72	0.91	0.81	-154.97	0.00*	0.18	0.91	9.76	0.51	-0.62	0.50	23.76	0.63	3.15	0.39	0.37	0.85	---	---

B = regression coefficient for predictor variables. All models are adjusted for age, gender, packyears, BMI, LDL cholesterol, and triglycerides. * significant *p*-value after Benjamini Hochberg correction for multiple testing

*CEL* N^ε^-(carboxyethyl)lysine, *CML* N^ε^-(carboxymethyl)lysine, *sRAGE* soluble receptor for advanced glycation endproducts, *AGE* advanced glycation end products, *RAGE* receptor for advanced glycation endproducts, *SAF* skin autofluorescence

### Genetic regulation of AGER gene expression levels and RAGE protein levels in sputum

**Fig. 5:**
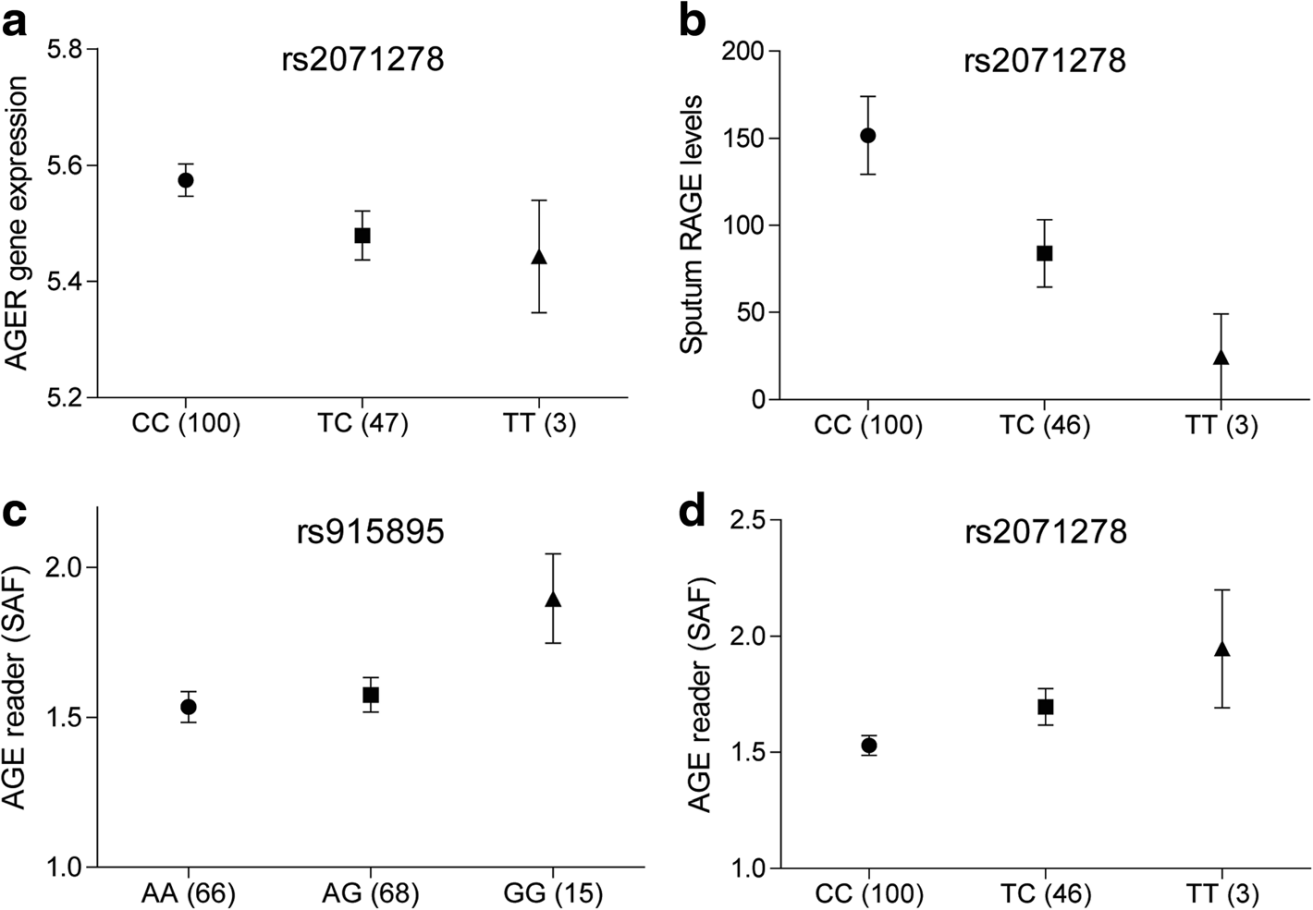
Genetic regulation of AGER gene expression levels, RAGE protein levels in sputum and AGE levels in skin. **a** Expression QTL analysis and **b** Protein QTL analysis of the AGER gene expression in bronchial biopsies and levels of soluble RAGE in sputum, respectively. SNP association study with AGE levels detected in the skin with **c** rs915895 and **d** rs2071278

Cis-eQTL analysis identified a single SNP (rs2071278) within a region of 50 kB flanking the AGER gene, which was associated with gene expression levels of AGER (beta = -0.109 ± 0.044, FDR < 0.25) (Fig. 5a, Additional file 1: Table S3). The SNP was located 13345 bp upstream from the promoter region of AGER. Protein QTL analysis (pQTL) was then run to investigate whether the rs2071278 eQTL influenced the levels of sRAGE detected in sputum and plasma. This analysis showed that rs2071278 regulated the levels of sRAGE measured in sputum (beta = -80.05 ± 30.80, *p* = 0.01), with the minor allele leading to decreased levels of sRAGE (Fig. 5b). No interaction was found between rs2071278 and sRAGE levels measured in plasma (beta = 47.62 ± 59.58, *p* = 0.425). Finally, we investigated whether levels of AGE accumulation in the skin were genetically regulated. A SNP association analysis was conducted on the SAF levels. From this analysis a single SNP was identified rs915895 (0.110 ± 0.038, FDR < 0.25), 38118 bp upstream of the AGER gene, which is significantly associated with AGE accumulation in the skin (Fig. 5c, Additional file 1: Table S4). Of interest, rs2071278 has a trend for significance (beta = 0.089 ± 0.049, *p* = 0.072), with the minor allele leading to increased SAF levels in the skin (Fig. 5d).

## Discussion

In this study we investigated a large COPD and non-COPD control population with respect to the accumulation of AGEs and the expression of its receptor RAGE in different body compartments, including plasma, induced sputum, bronchial biopsies and the skin. COPD has been associated with chronic oxidative stress, the most important accelerator of AGE formation. Our study shows that SAF values in the skin were higher in COPD patients compared to young and old non-COPD controls, whereas the expression of AGEs in bronchial biopsies was not different between the groups. In addition, sRAGE levels in plasma were lower in COPD patients. Of interest, lower sRAGE was associated with higher SAF, fitting the hypothesis of a ‘protective’ function of sRAGE by acting as a decoy-receptor preventing accumulation of AGEs in the skin.

In COPD, oxidative stress is thought to be continuously increased as a consequence of ongoing inflammation (endogeneous component) and chronic smoking (exogeneous component). This continuous exposure to oxidative stress, both locally in lung tissue as well as systemically in peripheral blood, might lead to increased accumulation of AGEs inside and outside the lung. In the current study we demonstrated that the accumulation of AGEs was elevated in the skin of COPD patients, a finding that we and others have observed before [18, 19]. Interestingly, SAF values did not differ significantly between the different severity stages of COPD. This suggests that the formation of AGEs is not increased during disease progression, but may be accelerated in the induction phase of COPD. Our data suggests that AGEs accumulate to some extent during ageing due to oxidative stress responses, whereas this is accelerated with smoking. The highest levels of AGEs would be expected in ‘susceptible’ smokers who develop COPD over time. Here, AGEs accumulate due to a combination of ageing and disease-related exaggerated responses to smoking and associated local and systemic oxidative stress. Together, our data shows that skin autofluorescence is significantly increased in COPD patients, without significant differences between the different severity stages of COPD.

In contrast with our findings in the skin, the expression of AGEs in bronchial biopsies was not different between COPD patients and non-COPD controls and did not associate with lung function values in the total population. This contradicts a previous study showing higher expression of AGEs in the lung parenchyma and small airways of COPD patients as compared to non-COPD controls [9]. In an effort to replicate these findings we analyzed immunopositivity of our bronchial biopsies in several ways, e.g. by quantifying AGEs in different parts of the bronchial biopsies and by using different antibodies (against total amount of AGEs, CML and pentosidine). However, no differential expression between the groups was observed. There are several explanations for our negative findings in bronchial biopsies. First, we collected biopsies from the central airways whereas oxidative stress might predominantly exist in the peripheral airways**,** and thereby also the formation of AGEs. Unfortunately, studies comparing oxidative stress in central and peripheral airways are scarce. One study showed that isoprostane levels in epithelial lining fluid (ELF) from the peripheral airways were higher compared to the central airways, both in smokers with and without airway obstruction [23]**.** Furthermore, we measured the expression of AGEs in peripheral lung tissue sections of smokers and non-smokers with and without COPD but did not find differences between these (small) groups. Secondly, accumulation of AGEs in the lung might be limited because of the relatively high turn-over rate of cells and extracellular matrix [24]. Finally, a quantification problem may contribute to a lower expression of AGEs in the lungs of COPD patients, as extracellular matrix proteins are reduced in the central airways of COPD patients [25]. Obviously, more research aimed at the accumulation of AGEs is needed in both the central and peripheral airways before definitive conclusions can be drawn regarding our conflicting results.

In plasma, we demonstrated that CML, CEL and pentosidine levels were comparable between COPD patients and non-COPD controls, after correction for confounding factors. Our results are in line with three previous studies in COPD investigating plasma CML levels and showing no differences between COPD and non-COPD controls [14, 16, 26]. In contrast, one study showed similar pentosidine levels, but lower CML levels and higher CEL levels in COPD patients [19]. The latter is surprising since CML and CEL are both formed by the same pathway, namely via reactive carbonyl compounds. One explanation might be that the quantification was performed using a different technique, i.e. mass spectrometry. Moreover, AGEs are very volatile, hence a measurement represents a ‘snap shot’ in time and results can be affected by food intake and smoking as well [1, 27].

Besides their harmful local effects in tissue, AGEs can interact with RAGE, thereby triggering intracellular signaling in pro-inflammatory pathways. Two previous studies showed that immunostaining of RAGE was increased in bronchial biopsies and in lung parenchyma of COPD patients [9, 10]. However, we observed no differences when comparing COPD with non-COPD controls in the current study. RAGE also exists as a soluble form, generated as a splice variant of the advanced glycosylation end product-specific receptor (*AGER*) gene or by proteolysis of the receptor from the cell surface. In line with previous studies [13–17], we demonstrated lower levels of sRAGE in plasma of COPD patients and these reduced levels were associated with lower lung function values in the total population. Importantly, we demonstrated for the first time that lower sRAGE levels were associated with increased SAF values, indicating that sRAGE acts as a decoy receptor. The binding of AGEs to sRAGE may induce the clearance of AGEs, thereby preventing the accumulation in body tissues. There are indications that lower sRAGE levels are genetically determined, as a single nucleotide polymorphism (SNP) in the *AGER* gene associates with lower sRAGE levels [13]. In this perspective, impaired sRAGE levels might contribute to higher levels of AGEs in tissues specifically in COPD.

Furthermore, we assessed the levels of AGEs and RAGE in sputum supernatant, which has not been studied in COPD before. We hypothesized that the sputum levels of AGEs and RAGE might reflect their expression in the lung. No differential levels of both AGEs and RAGE in induced sputum from COPD patients and non-COPD controls were found, nor associations with expression in bronchial biopsies. This observation fits with the similar expression of AGEs and RAGE between COPD and healthy individuals as observed in our bronchial biopsies. An explanation may be that AGEs are released in more peripheral airways and are not captured in sputum, as one study in COPD demonstrated that CML was elevated in ELF collected in the peripheral airways, but not in the central airways [28].

Finally, we have identified a novel SNP, rs2071278, which regulates both gene and protein expression of RAGE. Moreover, we also identified a SNP, rs2071278, showing a reversed association with the accumulation of AGEs in the skin. Further studies are planned to further elucidate the function of this SNP in the regulation of RAGE expression and its role in COPD patients.

This study is unique because of its large population of healthy smokers and never-smokers and a large group of COPD patients of all severities, as well as the availability of different tissues from each participant. In this study, only for 12 out of the 97 COPD patients sputum samples and bronchial biopsies were available, which limited the statistical power of this part of the study. However, even in these 12 patients we have shown a clear trend (*p* = 0.064) for CEL levels in induced sputum to be higher in COPD patients compared to both young and old healthy controls (Table 2). Another limitation is that our study had a cross-sectional design. Longitudinal studies are needed to investigate changes in AGEs and RAGE levels in the different tissues over time and to further assess the potential contributing role of AGEs and RAGE in the development of COPD. The accumulation of AGEs in the skin was assessed using the non-invasive AGE-reader (DiagnOptics Technologies B.V., Groningen, The Netherlands), which has shown to produce reliable values that strongly correlate with the levels of several non-auto-fluorescent AGEs measured in skin biopsies [21, 22]. However, there are some limitations to this technique, as the AGE-reader is unable to quantify specific AGEs and only estimates the group value of all AGEs present in the skin. Furthermore, also non-AGE-related auto-fluorescent proteins present in the skin can be detected by the AGE-reader.

## Conclusions

To summarize, the role of the AGEs-RAGE pathway in COPD is emerging. Our study contributes to this insight since we show an increased accumulation of AGEs in the skin of COPD patients compared to non-COPD smokers and never-smokers. Moreover, we did not observe differences between COPD and non-COPD controls in central bronchial biopsies, indicating that accumulation of AGEs is not similar in different body compartments. No further associations were found between AGEs and RAGE in the different compartments that were investigated. Interestingly, we demonstrated that lower sRAGE levels associate with higher AGE accumulation in the skin. This fits the hypothesis of a ‘protective’ function of sRAGE by acting as a decoy-receptor preventing accumulation in the skin. Lastly, we identified novel SNPs associated with both the gene and protein expression of RAGE and the accumulation of AGEs in the skin.

## Additional file

Additional file 1:
**Supplementary methods.** Sputum induction, bronchial biopsies, skin auto-fluorescence. **Supplementary Table 1.** AGE and RAGE expression in young and old never-smokers and smokers, and COPD GOLD stages. **Supplementary Table 2.** Correlations of AGEs and RAGE between different compartments. **Supplementary Table 3.** Expression QTL analysis of AGER. **Supplementary Table 4.** SNP association with AGE levels detected in the skin. **Supplementary Figure 1.** Quantitative analyses of AGEs (left panel) and RAGE (right panel) expression in A) intact epithelium, B) basal epithelium, C) smooth muscle, D) connective tissue of bronchial biopsies. Intensity of staining was scored by a 4-points scale: 0=negative staining, 1=weak positive, 2=positive, and 3=strong positive. Horizontal bars represent median values. **Supplementary Figure 2.** Representative immunohistological staining of AGEs (AGE 1/750, Cosmo Bio Clone 6D12) and RAGE (RAGE 1/1500, Abcam, ab7764) in young healthy controls, old healthy controls and COPD patients. Pictures are shown as 40x magnification, scans taken using the Hamamatsu Slide Scanner (Hamamatsu Photonics, Hamamatsu City, Japan). **Supplementary Figure 3.** Quantitative analyses of AGEs (left panel) and RAGE (right panel) expression in A) epithelium and B) smooth muscle of the peripheral airways. Intensity of staining was scored by a 4-points scale: 0=negative staining, 1=weak positive, 2=positive, and 3=strong positive. Horizontal bars represent median values.
