# Supplementary material for: Advanced glycation endproducts and their receptor in different body compartments in COPD
Source: Respir Res. 2016 Apr 26;17:46. doi: 10.1186/s12931-016-0363-2 (PMC4847335; doi:10.1186/s12931-016-0363-2)
Supplement: Additional file 1: — Supplementary methods. Sputum induction, bronchial biopsies, skin auto-fluorescence. Supplementary Table 1. AGE and RAGE expression in young and old never-smokers and smokers, and COPD GOLD stages. Supplementary Table 2. Correlations of AGEs and RAGE between different compartments. Supplementary Table 3. Expression QTL analysis of AGER. Supplementary Table 4. SNP association with AGE levels detected in the skin. Supplementary Figure 1. Quantitative analyses of AGEs (left panel) and RAGE (right panel) expression in A) intact epithelium, B) basal epithelium, C) smooth muscle, D) connective tissue of bronchial biopsies. Intensity of staining was scored by a 4-points scale: 0=negative staining, 1=weak positive, 2=positive, and 3=strong positive. Horizontal bars represent median values. Supplementary Figure 2. Representative immunohistological staining of AGEs (AGE 1/750, Cosmo Bio Clone 6D12) and RAGE (RAGE 1/1500, Abcam, ab7764) in young healthy controls, old healthy controls and COPD patients. Pictures are shown as 40x magnification, scans taken using the Hamamatsu Slide Scanner (Hamamatsu Photonics, Hamamatsu City, Japan). Supplementary Figure 3. Quantitative analyses of AGEs (left panel) and RAGE (right panel) expression in A) epithelium and B) smooth muscle of the peripheral airways. Intensity of staining was scored by a 4-points scale: 0=negative staining, 1=weak positive, 2=positive, and 3=strong positive. Horizontal bars represent median values. [file 12931_2016_363_MOESM1_ESM.doc]

**Advanced glycation endproducts and their receptor in different body compartments in COPD**

Susan J.M. Hoonhorst, Adèle T. Lo Tam Loi, Simon D. Pouwels, Alen Faiz, Eef D. Telenga, Maarten van den Berge, Leo Koenderman, Jan-Willem J. Lammers, H. Marike Boezen, Antoon van Oosterhout, Monique E. Lodewijk, Wim Timens, Dirkje S. Postma, Nick H.T. ten Hacken

**Supplementary Data**

**METHODS**

**Sputum induction**

Sputum induction was performed according to the method described elsewhere with some modifications (1). In short, 4,5% hypertonic saline was nebulized with an ultrasonic nebulizer (Ultraneb, DeVillbiss, Somerset, PA, USA). Patients inhaled for three periods of five minutes and were encouraged to cough and expectorate sputum after each period. The volume of the whole sputum sample was determined and an equal volume of 0.1% dithiothreitol (Sputolysin; Calbiochem, La Jolla, CA, USA) was added. The samples were agitated during 15 minutes in a shaking water bath for 15 minutes at 37°C to complete homogenization and then filtered through a 48 µm nylon gauze. The filtered sample was centrifuged (10 min, 450g, 4°C) and the supernatant was stored at -80°C until analysis.

**Bronchial biopsies**

After administering of local anaesthesia (lidocain 2-4%), a flexible bronchoscope was introduced and bronchial biopsies were taken from subsegmental carinae of the right lower lobe. Biopsies were fixed in 4% neutral buffered formalin, processed and embedded in paraffin and cut in 3 µm sections. Quality of biopsies was verified by hematoxylin and eosin (HE) staining. Sections were deparaffinized in xylene (2x10 min), rehydrated in alcohol dilations (2x100%, 2x96%, and 1x70%), and rinsed in demi-water. For AGEs staining, antigens were retrieved by incubating the slides in 0,1M Tris-HCl pH 9.0 buffer at 80°C overnight. For RAGE staining, Citrate 10mM pH 6.0 buffer was preheated, slides were placed in a plastic container and were heated in microwave for 15 min at 400W. After antigen retrieval the slides were cooled down at room temperature (RT) and were washed with PBS. All sections were incubated with 0.3% hydrogen peroxide H_2_O_2_ (Merck, Germany) in PBS (500µl H_2_O_2_ 30% in 50ml PBS) for 30 min at RT to block endogenous peroxidase activity. After three washes with PBS, sections were incubatjhed with the primary monoclonal antibody against AGEs (anti-AGEs (clone 6D12), 1:750, Cosmo Bio Co, Ltd, Tokyo, Japan) or RAGE (anti-RAGE (ab7764), 1:1500, Abcam, Cambridge, UK) diluted in PBS/1%BSA for 1 hour at RT. For AGEs staining, sections were washed in PBS for three times and incubated with the secondary antibody (Envision^TM^ Detection Systems Peroxidase (DAKO)) for 30 min at RT. For RAGE staining, sections were washed in PBS for 3 times and incubated with the secondary peroxidase labeled rabbit anti-goat antibody (DAKO, 1:100 diluted in PBS/1% BSA + 1%AB serum) for 30 min at RT. After washing with PBS 3x, sections were incubated with the tertiary peroxidase labeled goat anti-rabbit antibody (DAKO, 1:100 diluted in PBS/1% BSA + 1%AB serum) for 30 min at RT. After washing the sections for three times in PBS for three times, peroxidase activity was visualised by incubating the slides in DAB (3-3’DiaminoBenzidine) together with 50 µl of hydrogen peroxide for 10 min at RT. Sections were rinsed in demi water. Finally, the sections were counterstained with haematoxilin for approximately 2 min, rinsed in tap water, dehydrated in alcohol (70%, 96% and 100%), dried, and mounted with mounting medium and covered with a coverslip. Both immunohistochemical stainings were performed using the DAKO autostainer (DAKO, Glostrup, Denmark). Quantification of both stainings was performed by calculating the percentage positive and strong positive pixels of the total amount of pixels in whole biopsies, using ImageScope (Aperio Technologies, version 11.2.0.780).

**Skin autofluorescence**

SAF was assessed non-invasively by the AGE-Reader^TM^ (DiagnOptics B.V., Groningen, The Netherlands) (2). Technical details of this device have been extensively described elsewhere (3). In short, the AGE reader illuminates approximately 1 cm^2^ of the skin, guarded against surrounding light, with an excitation light source between 300 and 420 nm (peak excitation flow ~350 nm). Only light from the skin is measured between 300 and 600 nm with a spectrometer using a 200-µm glass fiber. SAF was calculated by dividing the average light intensity emitted per nm over the 420- to 600-µm range by the average light intensity emitted per nm over the 300- to 420-µm range, using the AGE Reader software version 2.2. The volar surface of subject’s forearm was positioned on top of the device, taking care to perform the measurement at normal skin site, i.e. without visible vessels, scars, or other skin abnormalities. SAF was averaged from three consecutive measurements for each subject, measured within a time period of approximately 2 minutes. In all analyses, SAF is expressed in arbitrary units (AU).

**SUPPLEMENTAL DATA**

**Table 1. AGE and RAGE expression in young and old never-smokers and smokers, and COPD GOLD stages**

**A. Young healthy never-smokers and smokers**

|  | **Young healthy never-smokers** | **Young healthy smokers** |
| --- | --- | --- |
| **Plasma** | *n=36* | *n=69* |
| CEL | 9.0 (6.7-15.0) | 11.4 (8.2-16.2) |
| CML | 10.9 (0.0-25.3) | 10.5 (0.0-38.1) |
| Pentosidine | 48.7 (32.6-70.9) | 35.3 (18.7-47.8)* |
| RAGE | 878.8 (573.3-1113.5) | 777.0 (628.8-1077.1) |
| **Sputum** | *n=34* | *n=63* |
| CEL | 4.0 (0.0-9.5) | 6.4 (3.8-8.2) |
| CML | 14.8 (0.0-44.8) | 0.0 (0.0-17.6)* |
| Pentosidine | 0.0 (0.0-0.0) | 0.0 (0.0-0.0) |
| RAGE | 0.0 (0.0-97.1) | 101.3 (0.0-195.1)* |
| **Bronchial biopsies** | *n=32* | *n=53* |
| AGEs, positivity (%) | 31.3 (18.5-37.4) | 30.6 (20.7-36.9) |
| RAGE, positivity (%) | 8.8 (6.1-14.8) | 10.8 (6.8-15.2) |
| **Skin** | *n=36* | *n=71* |
| AGE-reader | 1.20 (1.04-1.40) | 1.3 (1.1-1.5) |

**B. Old healthy never-smokers and smokers**

|  | **Old healthy never-smokers** | **Old healthy smokers** |
| --- | --- | --- |
|  | median (IQR) | median (IQR) |
| **Plasma** | *n=28* | *n=54* |
| CEL | 5.37 (3.29-8.55) | 8.56 (5.06-11.26)* |
| CML | 10.97 (0.00-13.64) | 0.00 (0.00-12.00) |
| Pentosidine | 39.53 (28.29-45.51) | 41.1 (30.12-66.48) |
| RAGE | 810.8 (672.9-1118.5) | 795.6 (609.3-953.3) |
| **Sputum** | *n=23* | *n=50* |
| CEL | 0.00 (0.00-3.23) | 6.11 (2.47-8.97)* |
| CML | 10.40 (0.00-23.49) | 11.46 (0.00-24.41) |
| Pentosidine | 0.00 (0.00-0.00) | 0.00 (0.00-0.00) |
| RAGE | 115.2 (0.0-206.9) | 114.0 (0.0-262.2) |
| **Bronchial biopsies** | *n=26* | *n=42* |
| AGEs, positivity (%) | 28.2 (19.1-37.4) | 25.4 (21.3-33.7) |
| RAGE, positivity (%) | 6.9 (5.0-10.6) | 9.5 (6.3-12.5) |
| **Skin** | *n=28* | *n=55* |
| AGE-reader | 1.774 (1.43-2.00) | 1.80 (1.60-2.10) |

**C. COPD severities**

|  |  |  |  |  | **Kruskall-Wallis** |
| --- | --- | --- | --- | --- | --- |
|  | **COPD GOLD I** | **COPD GOLD II** | **COPD GOLD III** | **COPD GOLD IV** | p-value |
| **Plasma** | *n=32* | *n=23* | *n=24* | *n=16* |  |
| CEL | 6.61 (4.92-9.05) | 5.37 (3.67-7.48) | 8.23 (5.92-9.58) | 7.34 (5.94-13.39) | 0.084 |
| CML | 13.76 (0.00-23.31) | 9.25 (0.00-18.70) | 13.06 (0.00-45.76) | 14.47 (0.00-21.66) | 0.508 |
| Pentosidine | 45.61 (30.29-59.85) | 49.17 (30.28-58.88) | 39.63 (30.50-49.28) | 53.67 (39.81-66.58) | 0.111 |
| RAGE | 510.02 (397.75-672.20) | 423.9 (372.3-635.8) | 314.40 (249.45-500.79)^§^ | 295.8 (216.5-412.2)^§‡^ | **0.001*** |
| **Sputum** | *n=0* | *n=10* | *n=2* | *n=0* |  |
| CEL | - | 13.75 (nvt) | 13.75 (nvt) | - | 0.086 |
| CML | - | 18.61 (nvt) | 18.6 (nvt) | - | 0.83 |
| Pentosidine | - | 0.00 (0.00-0.00) | 0.00 (0.00-0.00) | - | 1,000 |
| RAGE | - | 110.9 (77.4-176.3) | 74.3 (nvt) | - | 0.667 |
| **Bronchial biopsies** | *n=0* | *n=10* | *n=2* | *n=0* |  |
| AGEs, positivity (%) | - | 23.6 (15.9-31.7) | 26.7 (25.1-26.7) | - | - |
| RAGE, positivity (%) | - | 7.3 (3.3-11.0) | 12.4 (5.7-12.4) | - | - |
| **Skin** | *n=32* | *n=25* | *n=24* | *n=15* |  |
| AGE-reader | 2.42 (2.20-2.86) | 2.47 (2.03-2.95) | 2.71 (2.20-3.14) | 2.51 (2.17-2.89) | 0.612 |

CEL= N^ε^-(carboxyethyl)lysine, CML= N^ε^-(carboxymethyl)lysine, RAGE= receptor for advanced glycation endproducts, AGEs= advanced glycation endproducts, SAF= skin autofluorescence.

**Table 2. Correlations of AGEs and RAGE between different compartments**

|  |  | **Plasma** | |  |  |  |  | **Sputum** | |  |  |  |  |  |  | **Bronchial biopsies** | | |  | **Skin** |  |
| --- | --- | --- | --- | --- | --- | --- | --- | --- | --- | --- | --- | --- | --- | --- | --- | --- | --- | --- | --- | --- | --- |
|  |  | CML |  | Pentosidine | | RAGE |  | CEL |  | CML |  | Pentosidine | | RAGE |  | AGEs positivity | | RAGE positivity | | SAF |  |
|  |  | *Rho* | *p* | *Rho* | *p* | *Rho* | *p* | *Rho* | *p* | *Rho* | *p* | *Rho* | *p* | *Rho* | *p* | *Rho* | *p* | *Rho* | *p* | *Rho* | *p* |
| **Plasma** | CEL | **0.12** | **0.05** | **-0.12** | **0.06** | 0.10 | 0.12 | **0.28** | **<0.01** | 0.07 | 0.38 | 0.09 | 0.25 | -0.08 | 0.30 | 0.09 | 0.28 | **0.16** | **0.05** | **-0.29** | **<0.01** |
|  | CML | --- | --- | 0.05 | 0.41 | **-0.22** | **<0.01** | **0.16** | **0.03** | 0.01 | 0.92 | -0.01 | 0.90 | -0.01 | 0.94 | **0.21** | **0.01** | -0.00 | 0.99 | 0.04 | 0.55 |
|  | Pentosidine | --- | --- | --- | --- | **-0.16** | **0.01** | 0.05 | 0.54 | 0.00 | 0.99 | -0.03 | 0.67 | -0.06 | 0.45 | 0.13 | 0.10 | -0.05 | 0.52 | **0.12** | **0.05** |
|  | RAGE | --- | --- | --- | --- | --- | --- | **-0.15** | **0.06** | 0.00 | 0.97 | 0.01 | 0.94 | 0.06 | 0.46 | **-0.17** | **0.03** | -0.09 | 0.28 | **-0.46** | **<0.01** |
| **Sputum** | CEL | --- | --- | --- | --- | --- | --- | --- | --- | **0.26** | **<0.01** | 0.10 | 0.18 | 0.04 | 0.56 | **0.20** | **0.02** | 0.12 | 0.15 | **-0.16** | **0.03** |
|  | CML | --- | --- | --- | --- | --- | --- | --- | --- | --- | --- | 0.02 | 0.83 | **-0.29** | **<0.01** | -0.07 | 0.38 | 0.07 | 0.38 | 0.11 | 0.15 |
|  | Pentosidine | --- | --- | --- | --- | --- | --- | --- | --- | --- | --- | --- | --- | **-0.19** | **0.01** | -0.08 | 0.56 | -0.10 | 0.26 | -0.10 | 0.18 |
|  | RAGE | --- | --- | --- | --- | --- | --- | --- | --- | --- | --- | --- | --- | --- | --- | -0.09 | 0.79 | -0.03 | 0.72 | 0.10 | 0.18 |
| **Bronchial biopsies** | AGEs positivity | --- | --- | --- | --- | --- | --- | --- | --- | --- | --- | --- | --- | --- | --- | --- | --- | **0.24** | **<0.01** | 0.06 | 0.44 |
|  | RAGE positivity | --- | --- | --- | --- | --- | --- | --- | --- | --- | --- | --- | --- | --- | --- | --- | --- | --- | --- | -0.12 | 0.14 |

Spearman’s rank correlations, *Rho*=correlation coefficient. CEL= N^ε^-(carboxyethyl)lysine, CML= N^ε^-(carboxymethyl)lysine, RAGE= receptor for advanced glycation endproducts, AGEs= advanced glycation endproducts, SAF= skin autofluorescence.

Table 3. Expression QTL analysis of AGER

| **SNP** | **beta** | **SD** | **pvalue** | **FDR** |
| --- | --- | --- | --- | --- |
| **rs2071278** | **-0.109** | **0.044** | **0.015** | **0.223** |
| rs3130349 | -0.076 | 0.039 | 0.055 | 0.83 |
| rs204991 | -0.072 | 0.042 | 0.086 | 1 |
| rs3134608 | -0.063 | 0.038 | 0.099 | 1 |
| rs915895 | -0.038 | 0.035 | 0.278 | 1 |
| rs2269424 | 0.043 | 0.041 | 0.290 | 1 |
| rs204995 | -0.039 | 0.037 | 0.299 | 1 |
| rs394657 | -0.027 | 0.036 | 0.457 | 1 |
| rs2256594 | 0.035 | 0.050 | 0.482 | 1 |
| rs415929 | 0.026 | 0.037 | 0.494 | 1 |
| rs483574 | 0.015 | 0.046 | 0.741 | 1 |
| rs1061808 | 0.011 | 0.033 | 0.741 | 1 |
| rs2071279 | 0.010 | 0.039 | 0.807 | 1 |
| rs2269425 | 0.007 | 0.045 | 0.872 | 1 |
| rs436388 | 0.003 | 0.036 | 0.924 | 1 |

Table 4. SNP association with AGE levels detected in the skin

| **SNP** | **beta** | **SD** | **p value** | **FDR** |
| --- | --- | --- | --- | --- |
| **rs915895** | **0.109864** | **0.037731** | **0.004167** | **0.0625** |
| rs3134608 | 0.084364 | 0.041123 | 0.04203 | 0.6304 |
| rs204995 | 0.083171 | 0.040609 | 0.042368 | 0.6355 |
| rs2071278 | 0.088659 | 0.048928 | 0.072067 | 1 |
| rs3130349 | 0.073468 | 0.043113 | 0.090527 | 1 |
| rs204991 | 0.077416 | 0.045642 | 0.092021 | 1 |
| rs2071279 | -0.05887 | 0.042409 | 0.167293 | 1 |
| rs2269424 | -0.06125 | 0.044409 | 0.169969 | 1 |
| rs415929 | -0.03414 | 0.041075 | 0.407249 | 1 |
| rs436388 | -0.03174 | 0.039232 | 0.419781 | 1 |
| rs394657 | 0.028533 | 0.039783 | 0.474401 | 1 |
| rs1061808 | -0.01271 | 0.036559 | 0.728537 | 1 |
| rs483574 | -0.01672 | 0.050279 | 0.73994 | 1 |
| rs2269425 | -0.0083 | 0.048829 | 0.865289 | 1 |
| rs2256594 | 0.001179 | 0.054917 | 0.982899 | 1 |

**Figure 1. Quantitative analyses of AGEs and RAGE expression in bronchial biopsies**

Quantitative analyses of AGEs (left panel) and RAGE (right panel) expression in A) intact epithelium, B) basal epithelium, C) smooth muscle, D) connective tissue of bronchial biopsies. Intensity of staining was scored by a 4-points scale: 0=negative staining, 1=weak positive, 2=positive, and 3=strong positive. Horizontal bars represent median values.

**Figure 2. Representative staining of AGEs and RAGE expression in bronchial biopsies**

**
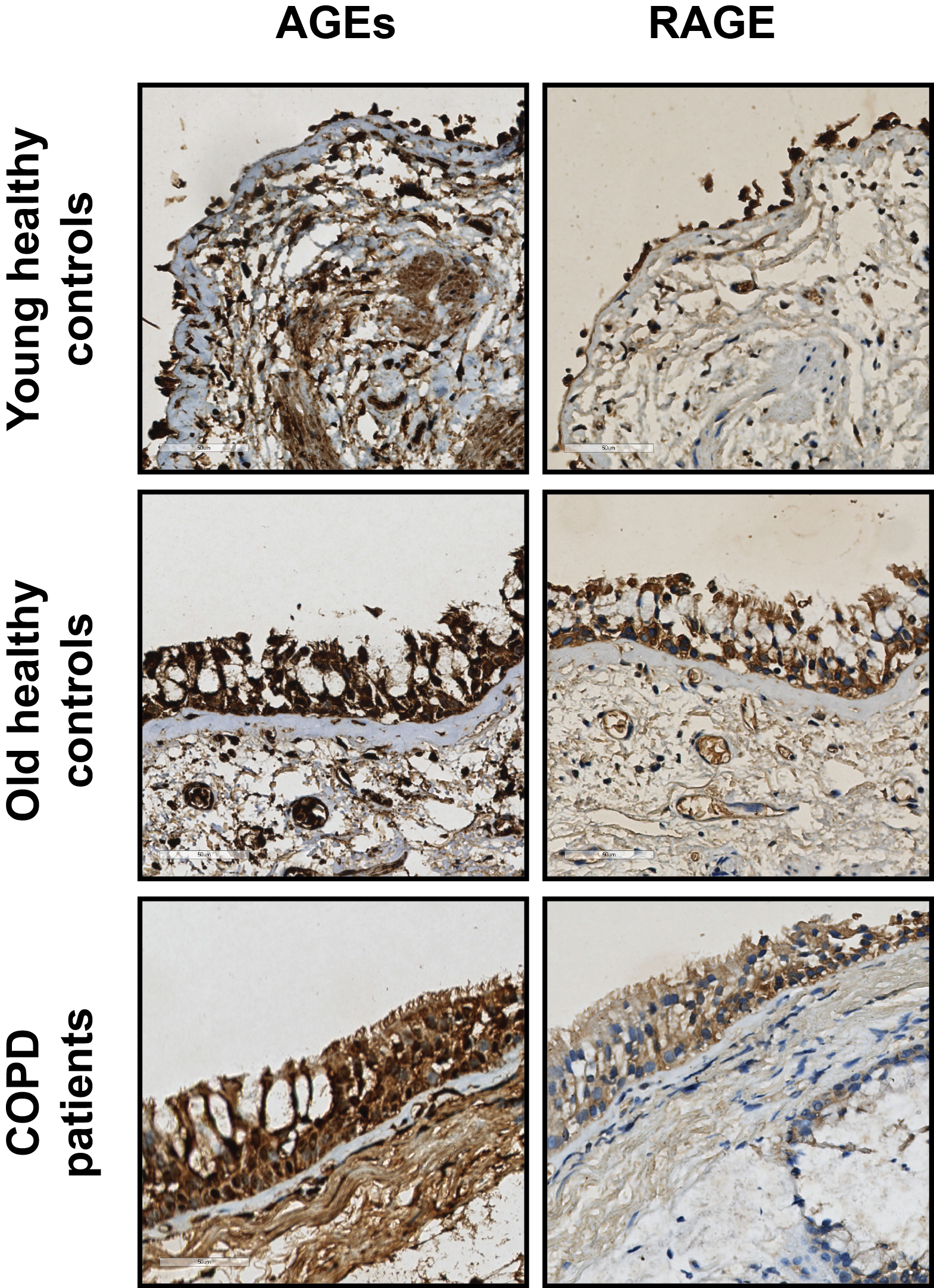
**

Representative immunohistological staining of AGEs (AGE 1/750, Cosmo Bio Clone 6D12) and RAGE (RAGE 1/1500, Abcam, ab7764) in young healthy controls, old healthy controls and COPD patients. Pictures are shown as 40x magnification, scans taken using the Hamamatsu Slide Scanner (Hamamatsu Photonics, Hamamatsu City, Japan).

**Figure 3. Quantitative analyses of AGEs and RAGE expression in peripheral airways**

Quantitative analyses of AGEs (left panel) and RAGE (right panel) expression in A) epithelium and B) smooth muscle of the peripheral airways. Intensity of staining was scored by a 4-points scale: 0=negative staining, 1=weak positive, 2=positive, and 3=strong positive. Horizontal bars represent median values.

**REFERENCES**

(1) Rutgers SR, Timens W, Kaufmann HF, van der Mark TW, Koeter GH, Postma DS. Comparison of induced sputum with bronchial wash, bronchoalveolar lavage and bronchial biopsies in COPD. *Eur Respir J* 2000;15:109-115.

(2) Meerwaldt R, Links T, Graaff R, Thorpe SR, Baynes JW, Hartog J, Gans R, Smit A. Simple noninvasive measurement of skin autofluorescence. *Ann N Y Acad Sci* 2005;1043:290-298.

(3) Meerwaldt R, Graaff R, Oomen PH, Links TP, Jager JJ, Alderson NL, Thorpe SR, Baynes JW, Gans RO, Smit AJ. Simple non-invasive assessment of advanced glycation endproduct accumulation. *Diabetologia* 2004;47:1324-1330.
